# T6 Treatment and Its Effects on Corrosion Properties of an Mg–4Sn–4Zn–2Al Alloy

**DOI:** 10.3390/ma11040628

**Published:** 2018-04-19

**Authors:** Xuefei Huang, Guomin Han, Weigang Huang

**Affiliations:** 1 College of Materials Science and Engineering, Sichuan University, Chengdu 610065, China; huangxf08@scu.edu.cn; 2 Institute of Applied Physics and Computational Mathematics, Beijing 100094, China; tianshuiyue@gmail.com; 3 CAEP Software Center for High Performance Numerical Simulation, Beijing 100088, China

**Keywords:** magnesium, precipitation hardening, alloying, corrosion rate

## Abstract

The effects of T6 treatment (solid-solution and artificially aging at 200 °C) on the microstructure and corrosion properties of an Mg–4Sn–4Zn–2Al (TZA442) alloy were systematically investigated. The alloy exhibits a double-peak age-hardening behavior, i.e., one is 78 HV after 10 h of aging, and the other is 83 HV after 50 h of aging. The strengthening effect is mainly attributed to the simultaneously and mutually independent precipitation of the dispersively distributed MgZn_2_ and Mg_2_Sn precipitates. Solid-solution treatment can significantly decrease the corrosion rate of the TZA442 alloy. The following aging treatment can initially further decrease the corrosion rate in the under-aged state, but can afterward slightly increase it after 50 h of aging. The relationship between the microstructure and corrosion properties is also discussed.

## 1. Introduction

As the lightest metallic structural materials, Mg alloys show great potential for applications in aerospace, transportation, and electrical industries. However, the poor corrosion resistance of many Mg alloys in aqueous environments has restricted their applications in many fields [1,2]. In addition, the strength of many Mg alloys is still unsatisfactory compared with their counterpart Al alloys [3]. Various processing methods, such as grain refining [4], deformation [5,6], and heat-treatment [7] have been employed to improve the mechanical properties of Mg alloys. Among these methods, the T6 treatment, i.e., solution and artificial aging treatment, is an effective and economical way of improving the strength of heat-treatable wrought Mg alloys [3]. Some Mg alloys can be significantly strengthened by the dispersively distributed fine precipitates resulting from T6 treatment [7]. However, since such aging treatment makes a significant modification on the microstructure of alloys, it may correspondingly affect the corrosion resistance. Therefore, it is vital that the microstructure evolution during heat-treatment and its effects on the corrosion resistance of Mg alloys be studied.

Mg–Sn–Zn–Al alloy is a recently developed wrought Mg alloy [8]. The alloy usually contains nearly equal amounts of Sn and Zn—amounts of over 4 wt % [9]. This is much different from the typical Mg alloys containing one major alloying element and several micro-alloying elements. For the processing route, the alloy is expected to be deformed in the solid-solution state, and then subjected to aging treatment to obtain better mechanical properties [1,10]. However, the aging behavior of the alloy has not been thoroughly studied. Though different precipitates have been found in the aged alloy and the corresponding mechanical properties has been characterized, the relationship between the precipitation process and age-hardening response has not been well established [11]. The multi-phase microstructure of the alloy containing different precipitates can be beneficial in terms of strengthening but will also significantly affect the corrosion properties. Since the very negative corrosion potential of α-Mg, the corrosion of the aged Mg alloys may be accelerated by the micro-galvanic coupling between anodic α-Mg phase and the precipitates [12]. On the other hand, it has been reported that the proper morphology and distribution of the precipitates may also act as barriers against corrosion propagation, such as the Mg_17_Al_12_ precipitates in Mg–Al alloys [13]. To the best of the authors’ knowledge, it is still unclear the effects of aging treatment on the corrosion resistance of Mg–Sn–Zn–Al alloys. Therefore, the present study has made a systematic investigation on the effects of T6 treatment (solid-solution and artificially aging at 200 °C) on the microstructure and corrosion properties of an Mg–4Sn–4Zn–2Al (denoted as TZA442) alloy. The aim of the study is to elucidate the relationship between the age-hardening response as well as the corrosion properties and the detailed multi-phase microstructures. The results can advance our knowledge on the strengthening and corrosion mechanism of TZA 442 alloy in terms of precipitate microstructures.

## 2. Experimental Procedure

### 2.1. Materials Preparation and Microstructure Characterization

An alloy with a nominal composition of Mg–4Sn–4Zn–2Al (in weight percent, wt %) was prepared in an electric furnace under protective atmospheres of CO_2_ and SF_6_, and then cast into a steel mold. In order to prevent possible melting of the different eutectics along grain boundaries, the homogenization of the cast alloy was carried out through a multi-step heating process, i.e., firstly hold at 300 °C for 4 h, then 420 °C for 24 h and finally solution treated at 460 °C for 2 h, followed by quenching into water. The aging treatment was then performed in a drying oven at 200 °C for various holding times. The age-hardening response was examined by a Vickers hardness tester under a load of 0.2 kg. Samples for optical microscopy (OM) were etched in a solution of acetic picral (1 mL of acetic acid, 1.2 g of picric acid, 2 mL of water, and 20 mL of ethanol). Phase constituents analysis was carried out in a Bruker DW-1000 X-ray diffraction (XRD) (Bruker Corporation, Billerica, MA, USA) machine. Transmission electron microscopy (TEM) (JEOL, Ltd., Tokyo, Japan) specimens were prepared by twin-jet electropolishing in a solution containing 3 mL of perchloric acid and 297 mL of ethanol. Characterization of precipitate microstructure was performed on a JEOL 2100F TEM. 

### 2.2. Corrosion Experiment

The corrosion behaviors were evaluated both by an immersion test and a potentiodynamic polarization test. The immersion test was carried out on cubic samples with dimensions of 10.5 mm × 10.5 mm × 10.5 mm. All the samples were firstly ground using SiC papers up to 2000 grit, and then cleaned with acetone. After that, the samples were weighed and then immersed in 3.5 wt % NaCl solution saturated with Mg(OH)_2_ for 48 h. During the immersion test, the temperature of the solution was maintained at a constant value of 25 °C. Subsequently, the samples were cleaned with a solution containing 20% CrO_3_ and 1% AgNO_3_ for 8 min to remove the corrosion products. Then the amount of weight loss was measured. The corrosion rates ***v*** were calculated using the following Equation (1).

(1) $$v=\frac{w_{1}-w_{0}}{S\cdot t}$$

where ***w*_0_** and ***w*_1_** is the weight (in mg) of the samples before and after the immersion, respectively. ***S*** is the total surface area in cm^2^, and ***t*** is the exposure time in hours. The potentiodynamic polarization test was carried out in a 3.5 wt % NaCl solution at 25 °C. The test was conducted in a conventional electrochemical cell with three electrodes of a working electrode (sample), a counter electrode (Pt plate), and a reference electrode (saturated calomel electrode, SCE) using a potentiostat (CHI 660E, Shanghai Chenhua Instrument Co., Ltd., Shanghai, China). For the polarization test, the samples were ground using SiC papers up to 2000 grit. The samples were then molded into epoxy resin with only one side of 1 cm^2^ exposed for the test. The potential sweep rate was 10 mV/s. 

## 3. Experimental Results

### 3.1. Optical Microstructure of TZA442 Alloy

**Figure 1:**
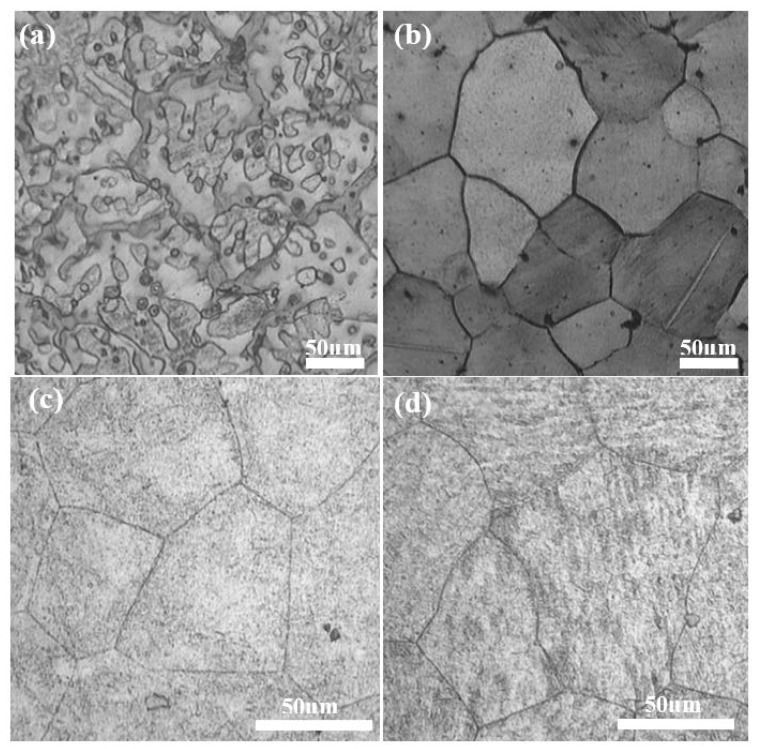
Optical microstructure of the TZA442 alloy: (**a**) the as-cast state; (**b**) the solution-treated state; (**c**) the 10-hour-aged state; (**d**) the 50-hour-aged state.

The optical microstructures of the investigated alloy in the as-cast and solution-treated state are shown in Figure 1a,b, respectively. The cast alloy exhibits a dendrite-like microstructure, which contains an α-Mg matrix and some secondary phases discontinuously distributed near the grain boundaries. These secondary phases were probably formed by the non-equilibrium solidification process. Some intragranular particles were also observed. Subsequent XRD analysis of the cast alloy shows that in addition to α-Mg, other peaks consistent with Mg_2_Sn and MgZn_2_ phases are also present, though the peaks are very weak. After the alloy was homogenized and solution-treated, most of the intermetallics are dissolved within the matrix (Figure 1b), which provides good conditions for the later aging treatment at 200 °C. After the homogenization and solid-solution process, the averaged grain size is around 100 μm. The OM microstructures with a higher magnification of the 10-hour-aged and 50-hour-aged TZA442 alloy are shown in Figure 1c,d, respectively. From the figures, many fine precipitates are found within the grains. As the aging time is prolonged, the number density of the precipitates is also increased. These precipitates are too fine to be resolved clearly by OM. It is noted that no clear intragranular blocky Mg_32_(Al,Zn)_49_ phases are observed in the solution-treated and aged TZA442 alloy. This is different from the results reported by Harosh et al. [9] and Zhang et al. [11]. In their work, both intergrannular and intragranular phases were observed in the solution-treated and aged Mg–Sn–Zn–Al alloy with a little higher Sn and Zn content. It is inferred that a little composition variation of Sn and Zn elements change the phase equilibrium of the quaternary TZA442 alloy.

### 3.2. Age-Hardening Behavior of TZA442 Alloy

**Figure 2:**
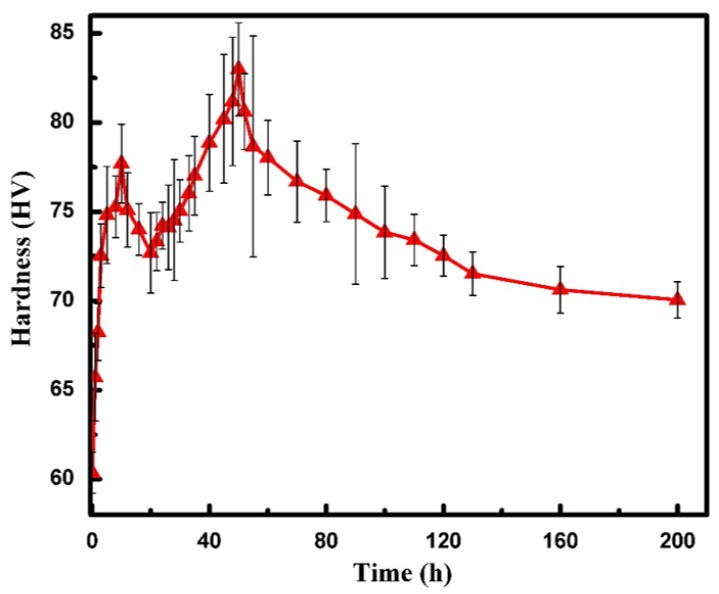
Age-hardening response of the TZA442 alloy.

After the alloy was solution-treated, the aging treatment was carried out at 200 °C. The age-hardening response of the TZA442 alloys is shown in Figure 2. The hardness value of the solution-treated alloy is about 61 HV. As the holding time increases, the hardness quickly increases to a local maximum value of 78 HV after 10 h. Then, during the later 10 h, a slight decrease in the hardness was detected. After that, the hardness increases again to reach a peak value of 83 HV in the 50th h. It then begins to decrease slowly to about 70 HV after 200 h of aging. Combined with the following XRD and TEM results, it can be inferred that the age-hardening response is associated with the precipitation of both the MgZn_2_ and Mg_2_Sn phases. However, the different aging kinetics of the two phases contributed to the double-peak aging behavior, which is different from the typical age-hardening response of Mg alloys subjected to a single aging at a constant temperature.

### 3.3. Precipitate Microstructure of the Aged Alloy

**Figure 3:**
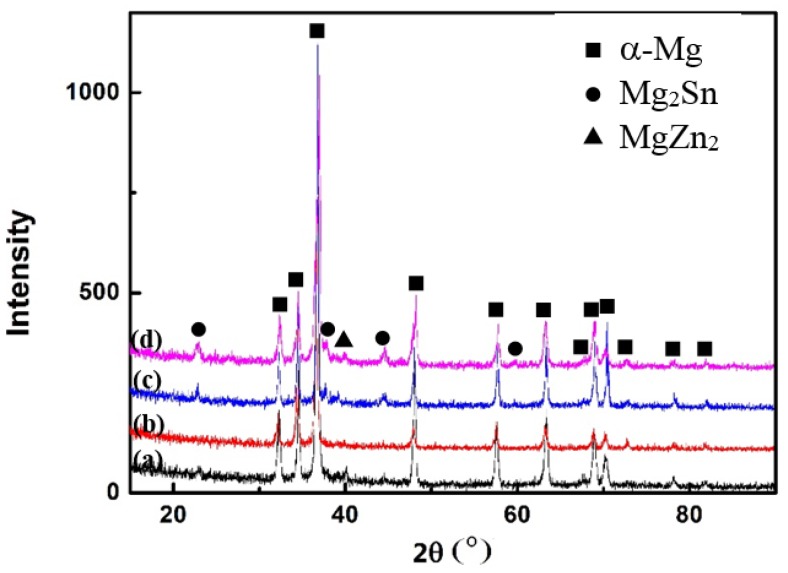
XRD patterns of the TZA442 alloy in different states: (**a**) the as-cast state; (**b**) the solid solution-treated state; (**c**) the 10-hour-aged state; (**d**) the 50-hour-aged state.

The change in the hardness indicates the precipitation process during the aging treatment. In order to investigate the phase constituents of the alloy, XRD experiments have been performed for the alloy in different states, as shown in Figure 3. For the cast TZA442 alloy, in addition to α-Mg, the peaks of the secondary phases are determined as Mg_2_Sn and MgZn_2_ phases. The peaks of the secondary phases are very weak, indicating that the amounts of the secondary phases are small. After the solution treatment, the peaks of both secondary phases disappeared. It can be inferred that most of the secondary phases were dissolved into the matrix. This is consistent with the previous OM results. After 10 h of aging at 200 °C, the peaks of the secondary phases appeared again. For the 10-hour-aged sample, the peaks of the Mg_2_Sn phases can be clearly detected, but the intensity from the peaks of MgZn_2_ is too weak. As the aging time increases to 50 h, the peak intensity of both precipitates becomes stronger, indicating that a large amount of Mg_2_Sn and MgZn_2_ are formed, which is also consistent with the previous OM results. No Al-containing phase has been detected, implying that Al probably exist as solute atoms in the Mg matrix. In order to further clarify the precipitate microstructure of the aged alloy, TEM characterizations were carried out. 

**Figure 4:**
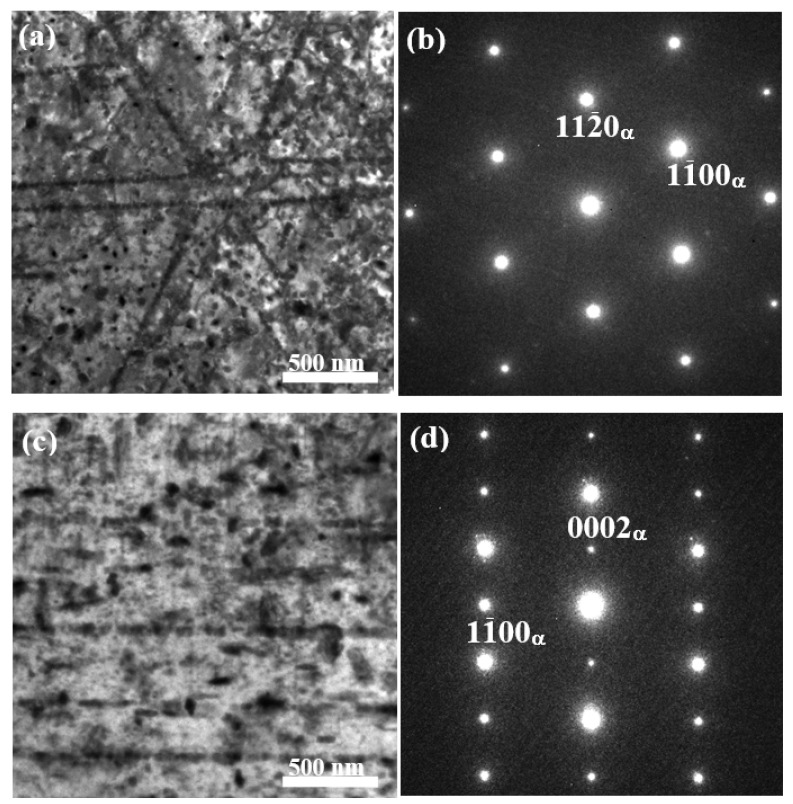
TEM micrographs showing the precipitate microstructures of the aged TZA442 alloy: (**a**) bright-field image obtained at the [0 0 0 1]_α_ zone axis and (**b**) the corresponding diffraction pattern; (**c**) bright-field image obtained at a <1 1 $\bar{2}$ 0>_α_ zone axis and (**d**) the corresponding diffraction pattern. ‘α’ in the lower right of the index indicate the α-Mg matrix.

Figure 4 provides the TEM micrographs showing the precipitate microstructure of the peak-aged TZA442 alloy. From the bright-field image obtained at [0 0 0 1]_α_ zone axis, a larger amount of precipitates were observed within the matrix. The precipitates have a size of tens of nanometers, and they are formed either homogeneously within the matrix or along the dislocation lines. Most of the precipitates exhibit small particle morphology and the anisotropic characteristic is not very obvious. From the bright-field image obtained near a <1 1 $\bar{2}$ 0>_α_ zone axis, it can be seen that some particles tend to lie on the basal (0 0 0 1)_α_ plane, while others are more apt to grow along the [0 0 0 1]_α_ direction. These nano-sized precipitates are believed to play an important role in the precipitation-hardening behavior of this alloy.

**Figure 5:**
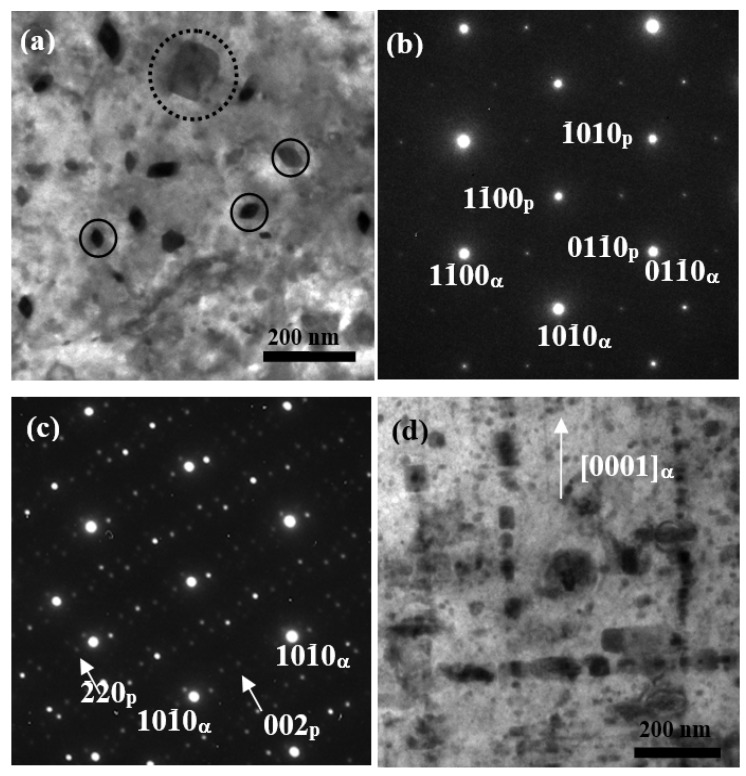
TEM micrographs showing the morphology and SAED patterns of the precipitates in the peak-aged TZA442 alloy. (**a**) Bright-field image showing the precipitate microstructure along the [0 0 0 1]_α_ zone axis; (**b**) superimposed diffraction patterns of the rhombic precipitates and the matrix, ‘p’ in the lower left of the index indicates the MgZn_2_ precipitate; (**c**) superimposed diffraction patterns of the plate precipitates and the matrix, with the extra spots from double diffractions, ‘p’ in the lower left of the index indicates theMg_2_Sn precipitate; (**d**) bright-field image showing the precipitate microstructure along a <1 $\bar{1}$ 0 0>_α_ zone axis.

In order to further identify the precipitates, the morphology and the selected area electron diffraction (SAED) patterns of the precipitates have been examined carefully. From the bright-field image obtained along the [0 0 0 1]_α_ zone axis shown in Figure 5a, the precipitates in the peak-aged TZ442 alloy exhibit mainly two different kinds of morphologies, i.e., one is the small precipitates showing a rhombic shape as indicated by solid circles, and the other is the large plate precipitates as indicated by the dotted circles. SAED patterns of the rhombic precipitates are shown in Figure 5b. By indexing the pattern of the precipitates, it is consistent with the [0 0 0 1] zone axis of MgZn_2_ phase, which has a hexagonal structure, with lattice parameters of a = 0.523 nm and c = 0.858 nm [14]. The orientation relationship between the MgZn_2_ precipitates and the matrix can be determined as [0 0 0 1]_MgZn2_//[0 0 0 1]_α_ and (1 1 $\bar{2}$ 0)_MgZn2_//(1 0 $\bar{1}$ 0)_α_. Superimposed diffraction patterns of the plate precipitates and the matrix are shown in Figure 5c. The indexed pattern of the precipitates is consistent with the [1 1 0] zone axis of the Mg_2_Sn precipitates, which adopted an orientation relationship of (1 1 0)_Mg2Sn_//(0 0 0 1)_α_ and [0 0 1]_Mg2Sn_//[1 1 $\bar{2}$ 0]_α_ with the α-Mg matrix. Both the crystallographic features of the two precipitates are consistent with that in the corresponding aged Mg–Zn [14] and Mg–Sn alloys [15], respectively. Therefore, it can be inferred that, for the current Mg–4Sn–4Zn–2Al alloy, the multi-alloying of Sn, Zn, and Al does not form any ternary phase, and the two major precipitates do not notably affect each other. In other words, the precipitation process of the MgZn_2_ and the Mg_2_Sn phases are mutually independent. From the magnified microstructure obtained at a <1 $\bar{1}$ 0 0>_α_ zone axis (Figure 5d), it can be clearly seen that the two distinct growing directions of the two types of precipitates. The Mg_2_Sn precipitates were determined to lie on the basal (0 0 0 1)_α_ plane, while the MgZn_2_ precipitates were found to grow along the [0 0 0 1]_α_ directions.

### 3.4. Corrosion Properties of TZA442 Alloy in Different States

**Figure 6:**
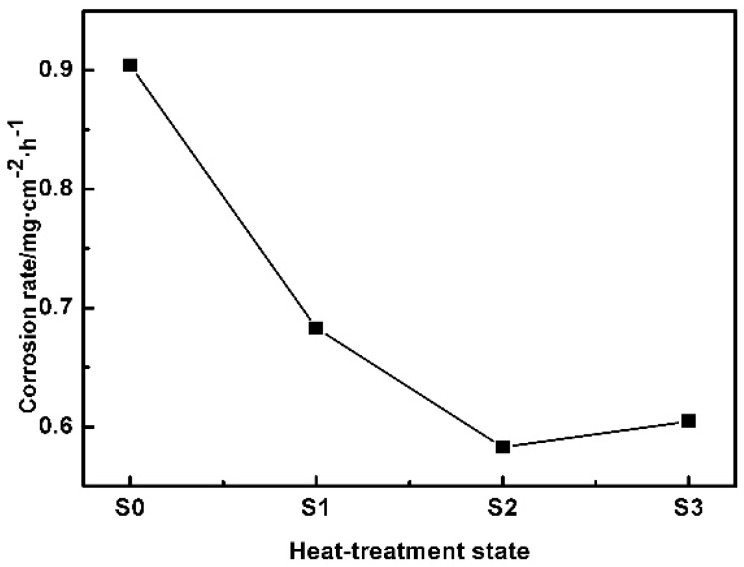
Corrosion rates of the TZA 442 alloy in different states by immersion test.

**Figure 7:**
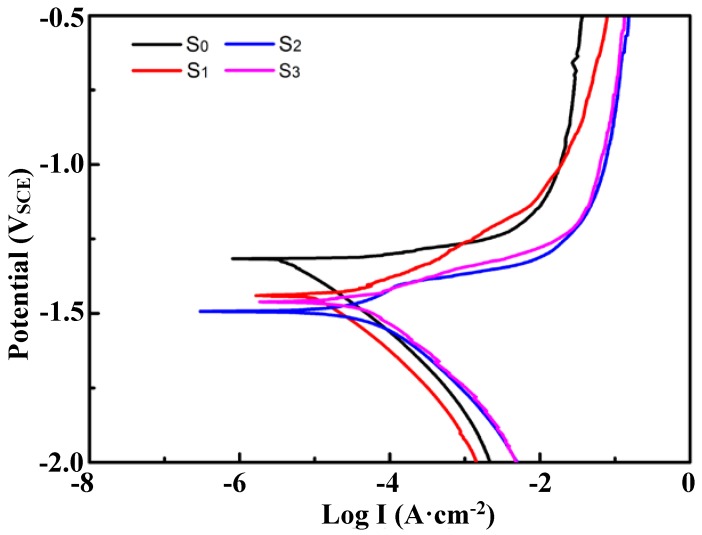
Potentiodynamic polarization curves of the TZA442 alloys in different states.

The alloys in the cast state (denoted as S0), α-Mg solution-treated state (denoted as S1), the 10-hour-aged state (denoted as S2), and α-Mg 50-hour-aged state (denoted as S3) were chosen to examine the effect of heat-treatment on the corrosion resistance. Figure 6 shows the weight loss rate of the alloys in different states for 48 h of immersion in corrosion solutions. The cast TZA442 alloy exhibits the largest corrosion rate of about 0.9 mg·cm^−2^·h^−1^. After the alloy was solution-treated, the corrosion rate decreases significantly to 0.69 mg·cm^−2^·h^−1^. It is interesting to note that after 10 h of aging, the corrosion rate further decreases to 0.59 mg·cm^−2^·h^−1^. However, when it was further aged to 50 h, the corrosion rate shows a slight increase to 0.60 mg·cm^−2^·h^−1^. The different corrosion rates indicate the different corrosion mechanism of the alloy in different states. Figure 7 shows the potentiodynamic polarization curves of the TZA442 alloy in different states. The polarization parameters measured from the polarization cures are summarized in Table 1. It shows that the cast alloy has a corrosion potential (*E_corr_*) of −1.32 V, and the maximum corrosion current density (*I_corr_*) of 10.5 × 10^−5^ A·cm^−2^. After the alloy was solution-treated, the corrosion potential decreased to −1.44 V, indicating a greater corrosion tendency of the alloy. However, the corrosion current density of the solution-treated alloy is significantly reduced to 3.5 × 10^−5^ A·cm^−2^. As the alloy was under-aged, the corrosion potential does not change considerably, and the corrosion current density is further decreased to 2.7 × 10^−5^ A·cm^−2^ after 10 h of aging. When the alloy, held at 200 °C, was peak-aged after 50 h, it has a corrosion potential of only −1.46 V, and the measured corrosion current density is 4.5 × 10^−5^ A·cm^−2^. The variation trend of the corrosion current density for the alloy in different states is nearly consistent with the corrosion rate shown in Figure 6. The corrosion rate can also be calculated from the corrosion current density. They were calculated as 2.258, 0.75, 0.58, and 0.97 mm/year, respectively for the S0, S1, S2, and S3 samples. They are comparable but still much different from the weight loss data. It should be noted that the corrosion environments could be different for the two different methods, and the corrosion time used in the immersion test is still not long enough to represent all corrosion behavior in a year.

## 4. Discussion

### 4.1. The Age-Hardening Response of TZA442 Alloy

The age-hardening response of TZA442 alloy certainly corresponds to the precipitation of the secondary phases. However, the aging behavior is clearly different from that of Mg–Sn or Mg–Zn alloys, in which the content of the major alloying elements is much larger than other micro-alloying elements. Generally, in the age-hardening curves of the Mg–Sn or Mg–Zn alloys subjected to T6 (solid-solution + artificial aging) treatment, only one peak hardness value was detected [7]. However, in the present alloy, where Sn and Zn concentrations are comparable, two peaks are present. From the XRD and TEM experimental results, it can be seen that the major strengthening precipitates are mainly MgZn_2_ and Mg_2_Sn phases, which are the major precipitates observed in Mg–Zn and Mg–Sn alloys, respectively. No Mg–Sn–Zn ternary phases were detected. Therefore, the age-hardening response of TZA442 alloy can be suggested as the co-precipitation of two precipitates, and the precipitation processes of these two phases are mutually independent. However, the precipitation kinetics of the two phases differs significantly. From the calculated diffusion coefficients of Zn and Sn in dilute Mg alloys, it can be seen that Zn diffuses more quickly than Sn in the directions both parallel and normal to the (0 0 0 1)_α_ plane [16]. Therefore, it can be inferred that MgZn_2_ probably precipitates more quickly than Mg_2_Sn in TZA442 alloy. It also reflects the different age-hardening response of Mg–Zn and Mg–Sn alloys. For an Mg–8Zn alloy aged at 200 °C, it takes only 24 h to achieve peak hardness [17]. While for an Mg–7Sn alloy also aged at 200 °C, the time needed for peak-hardening is 140 h [18]. Thus, for the present TZA442 alloy, it is proposed that the first peak hardness at 10 h probably resulted mainly from the precipitation of MgZn_2_, though some Mg_2_Sn was also formed. For the second hardness peak, both Mg_2_Sn and MgZn_2_ contributed to strengthening effects. In the present study, alloying elements at a total of 10 wt % (4% Sn + 4% Zn + 2% Al) in Mg only yield a peak hardness value of 83 HV in the aged alloy, which is higher than that of 80.4 HV in the Mg–7Sn–1Ca-1Ag alloy [18]. This can be attributed to the much refined precipitate size, as compared to the precipitate microstructure. Moreover, the aging kinetics is enhanced, and the cost of the alloy is reduced.

### 4.2. Relationship between the Microstructure and the Corrosion Properties

Based on the corrosion test given in Section 3.4, the corrosion rate of the as-cast TZA442 alloy is much higher than those of alloys solution-treated or aged. This is associated with the coarse secondary phase distribution in the as-cast microstructures. As shown in the OM results (Figure 1a), the secondary phases are mainly coarse eutectics formed by the non-equilibrium solidification process. In addition, only a few solute atoms exist in the cast alloy. After the solution treatment, most of the intermetallic phases were dissolved into the matrix, which became greatly supersaturated with the solute atoms of Sn, Zn, and Al. As a result, the corrosion rates are decreased, which is probably caused by the reduced micro-galvanic corrosion effect. However, when the alloy was under-aged at 200 °C for 10 h, though there are some fine precipitates in the matrix, the corrosion rate was further reduced. In this sense, the finely distributed nano-sized precipitates are suggested to be beneficial to the corrosion properties of the alloy, which is much different from the coarse intermetallics in the as-cast state. However, when the alloy was further aged to the peak-aged state, the corrosion rate slightly increased, indicating that the amount of the precipitates is too high to ignore the micro-galvanic corrosion effect. It is believed that the detailed corrosion behavior may also be associated with the distribution of solute atoms within the α-Mg matrix, the corrosion potential of the precipitates, the grain sizes, and so on [19]. A further systematic study on the effects of these metallurgical factors on the corrosion behaviors of the TZA442 alloy can provide clear clues for the corresponding corrosion mechanism.

## 5. Conclusions

In summary, the solid-solution and age-hardening behavior and its effects on the corrosion resistance of TZA442 alloy have been investigated. Conclusions drawn are as follows:

- The cast TZA442 alloy contains a small amount of Mg_2_Sn and MgZn_2_ intermetallics along the grain boundaries. Multi-alloying of Sn, Zn, and Al does not form any ternary phases in TZA442 alloy;
- After a thorough solid-solution treatment and subsequent artificial aging at 200 °C, the hardness of the alloy firstly increases quickly to a local maximum value of 78 HV after 10 h of aging. After a slight decrease, the hardness increases again to reach a second peak value of 83 HV after 50 h of aging, followed by a clear over-aging behavior. The strengthening effects are mainly attributed to the co-precipitation and mutually independent precipitation process of the MgZn_2_ and Mg_2_Sn precipitates, which show different aging kinetics;
- Solid-solution treatment can significantly decrease the corrosion rate of TZA442 alloy. When the solution-treated alloy was subjected to artificial aging treatment, the corrosion rate is further decreased in the under-aged state, but then begins to increase slightly in the 50-hour-aged state. The corrosion behavior of the alloy in different states is closely associated with their multi-phase microstructure.

## Figures and Tables

**Table 1:** Corrosion potential (*E_corr_*) and the corrosion current density (*I_corr_*) for TZA442 alloy in different heat-treatment states.

Samples	S_0_	S_1_	S_2_	S_3_
*E_corr_* (V)	−1.32	−1.44	−1.49	−1.46
*I_corr_* (A·cm^−2^)	10.5 × 10^−5^	3.5 × 10^−5^	2.7 × 10^−5^	4.5 × 10^−5^
